# Evaluation of automated radiostereometric image registration in total knee arthroplasty utilizing a synthetic‐based and a CT‐based volumetric model

**DOI:** 10.1002/jor.25359

**Published:** 2022-05-27

**Authors:** Emil Toft Petersen, Tobias Dahl Vind, Jonathan Hugo Jürgens‐Lahnstein, Rasmus Christensen, Sepp de Raedt, Annemarie Brüel, Søren Rytter, Michael Skipper Andersen, Maiken Stilling

**Affiliations:** ^1^ University Clinic for Hand, Hip and Knee Surgery, Holstebro Central Hospital Holstebro Denmark; ^2^ Department of Clinical Medicine Aarhus University Aarhus Denmark; ^3^ AutoRSA Research Group, Orthopaedic Research Unit Aarhus University Hospital Aarhus Denmark; ^4^ Department of Biomedicine Aarhus University Aarhus Denmark; ^5^ Department of Orthopaedic Surgery Aarhus University Hospital Aarhus Denmark; ^6^ Department of Materials and Production Aalborg University Aalborg Denmark

**Keywords:** automated, image registration, radiostereometry, RSA, total knee arthroplasty

## Abstract

Radiostereometic analysis (RSA) is an accurate method for rigid body pose (position and orientation) in three‐dimensional space. Traditionally, RSA is based on insertion of periprosthetic tantalum markers and manual implant contour selection which limit clinically application. We propose an automated image registration technique utilizing digitally reconstructed radiographs (DRR) of computed tomography (CT) volumetric bone models (*autorsa‐bone*) as a substitute for tantalum markers. Furthermore, an automated synthetic volumetric representation of total knee arthroplasty implant models (*autorsa‐volume*) to improve previous silhouette‐projection methods (*autorsa‐surface*). As reference, we investigated the accuracy of implanted tantalum markers (*marker*) or a conventional manually contour‐based method (*mbrsa*) for the femur and tibia. The data are presented as mean (standard deviation). The *autorsa‐bone* method displayed similar accuracy of −0.013 (0.075) mm compared to the gold standard method (*marker*) of −0.013 (0.085). The *autorsa‐volume* with 0.034 (0.106) mm did not markedly improve the *autorsa‐surface* with 0.002 (0.129) mm, and none of these reached the *mbrsa* method of −0.009 (0.094) mm. In conclusion, marker‐free RSA is feasible with similar accuracy as gold standard utilizing DRR and CT obtained volumetric bone models. Furthermore, utilizing synthetic generated volumetric implant models could not improve the silhouette‐based method. However, with a slight loss of accuracy the autorsa methods provide a feasible automated alternative to the semi‐automated method.

## 1 INTRODUCTION

Knee radiostereometric analysis (RSA) can quantify rigid body pose (position and orientation) in three‐dimensional (3D) space utilizing a calibrated setup with two crossing X‐ray beams, which produce radiographic images from different views.^1^, ^2^ Owing to submillimeter accuracy and precision, the RSA technique is widely used to evaluate longitudinal fixation of hip and knee implants as an early surrogacy marker for later aseptic implant loosening.^3^, ^4^, ^5^ Originally, RSA utilized tantalum markers attached on the investigated implants before surgery and inserted into the periprosthetic bone during surgery. There are several downsides to implant marking including expense, need for new regulatory approval of the implants, and a possible influence on fixation.^6^ Next, a commercially available RSA method utilizing implant models was introduced and allowed implant tracking without implant‐embedded markers at the expense of a slight loss of accuracy.^6^, ^7^ Today, this method still requires insertion of periprosthetic tantalum markers and manual implant contour selection in RSA images. Although RSA is a recommended safety measure for fixation of new implants, the requirement for beads as bone reference and lack of full analysis automatization restricts a general use of RSA for monitorization of implant loosening.^1^, ^5^, ^8^

3D bone models obtained from computed tomography (CT) has proven to be accurate for two‐ to three‐dimensional (2D/3D) image registration and may replace periprosthetic tantalum markers as reference in RSA.^9^ CT scans can be conducted postoperatively and permit RSA migration analysis in any patient. 2D/3D image registration techniques often use intensity or gradient measures to match silhouette projections or digitally reconstructed radiographs (DRRs).^9^, ^10^, ^11^, ^12^, ^13^, ^14^, ^15^, ^16^, ^17^, ^18^ Consequently, these methods are highly dependent on the geometrical shape, the intensities, and contrast to withhold the high accuracy. Theoretically, the registration may be affected by radiopaque metal implants and removal of bone, in terms of reduced bone model geometry and registration information, during insertion of implant components.^12^ Previous attempts to replace the markers as reference have not demonstrated feasible results.^19^, ^20^

The current 2D/3D image registration techniques of implants are utilizing silhouette‐projections of triangulated surface models and do not reach the accuracy of the marker‐method.^7^, ^18^ A synthetic volumetric representation of the implant with constant voxel values within the surface‐shell may improve the image registration accuracy utilizing DRR registration.

The purpose of this In vitro study was to investigate the pose accuracy of implant components from total knee arthroplasty and tibial and femoral bone models from CT scans using silhouette‐projections and DRRs. As reference, we investigated the accuracy of implanted tantalum markers in the femur and tibia.

## 2 METHODS

This study utilized eight fresh‐frozen donor legs including the hemi‐pelvis; male:female ratio 1:1, ages 80–93 (mean 85 years). Relevant approvals were obtained from the Central Denmark Committee on Biomedical Research Ethics (case number: 1‐10‐72‐236‐19, issued November 21st, 2019), and the Data Protection Agency (case number: 1‐16‐02‐410‐19, issued December 2nd, 2019).

### 2.1 Preparation and surgical procedure

Before surgery, the knee of each specimen was CT scanned (SOMATOM Definition Flash; Siemens Healthcare) including 15 cm proximal and distal to the joint line. The scans were carried out using a standard protocol with axial slices at a peak voltage of 120 kVp and exposure of 183 mAs, slice thickness of 0.6 mm, slice increment of 1 mm and pixel spacing of 0.29 × 0.29 mm. The effective dose of the CT was estimated to 0.095 mSv. Subsequently, all specimens were disarticulated at the hip and ankle joints and the proximal femoral and distal tibial bone were dissected for soft tissue to ensure a rigid fixation of the specimen. Approximately 8–13 tantalum beads (X‐medics) were inserted through a 4 mm drill hole in the cortical bone of the distal femoral and proximal tibial bones using a bead gun (Kulkanon; Wennbergs Finmek AB). Beads were placed in a systematic pattern intending a wide‐spread 3D marker distribution. We used a standard operative total knee arthroplasty procedure according to the manufacturer's guidelines^21^ with an anterior midline incision and medial parapatellar arthrotomy. All specimens received the cemented (Palacos®R + G, Heraeus, Medial GmbH, 61273) Triathlon® Knee System (Stryker) for the femur, tibia, and patella in size ranges of four to six. One experienced knee arthroplasty surgeon performed the surgical procedures on all specimens.

### 2.2 Radiographic setup

**Figure 1:**
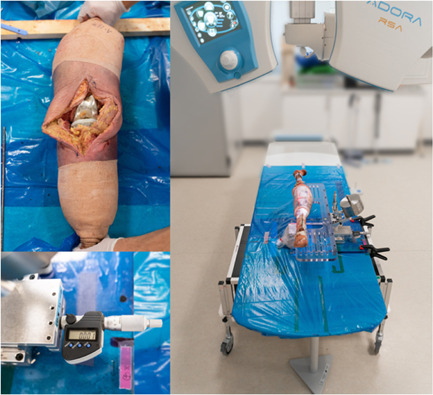
Illustration of the total knee arthroplasty knee before closure, the radiostereometric setup, and a closeup of the micrometer. [Color figure can be viewed at wileyonlinelibrary.com]

The stereoradiographs were recorded utilizing a dedicated RSA system (AdoraRSA; NRT X‐Ray A/S). The system uses two ceiling‐mounted X‐ray tubes positioned vertically with an inter‐tube angle of 40 degrees and a source‐to‐image distance of 160 cm. The flat panel detectors (CXDI‐50RF; Canon Inc.) were embedded in a uniplanar calibration cage (Box 24; Medis Specials) containing a fiducial and control layer. In single‐image mode, we acquired full detector‐size images dimensioned 2208 × 2668 pixels with a quadratic pixel width of 0.16 mm using exposure settings of 120 kVp and 1.2 mAs. The dose of one stereoradiograph was estimated to 0.623 µSv (Figure 1).

### 2.3 Experimental protocol

A customized fixture with an axial movable plexiglass plate was built and ensured rigid fixation of the knee specimen (Figure 1). Accommodating optimal radiographic imaging, a hole was cut out of the plexiglass at the knee level, ensuring free passage of the X‐ray beams. We moved the plate in three directions (*x*, *y*, *z*) using digital dial micrometers, each with a resolution of 0.001 mm (Hofmann GmbH). The fixture was oriented approximately orthogonal to the reference frame of the RSA setup (calibration cage). First, the knee was positioned anterior‐posterior (AP), replicating a patient in a supine position. Second, the knee was positioned in a lateral‐medial (LM) view to investigate influence of different view. Recordings were obtained in all directions (*x*, *y*, *z*) at 16 positions. Each series included five recordings at baseline. Second, two recordings were obtained each at 10, 20, 30, 40, 50, 100, 200, 300, 400, 500, 1000, 2000, 3000, 4000, 5000 μm. The corresponding measured displacement was established by the difference between the median of the estimated positions at baseline and the corresponding estimated position. The error was determined by subtracting the actual (micrometer [μm]) displacement from measured (RSA) displacement.

### 2.4 Analysis of the stereoradiographs

**Figure 2:**
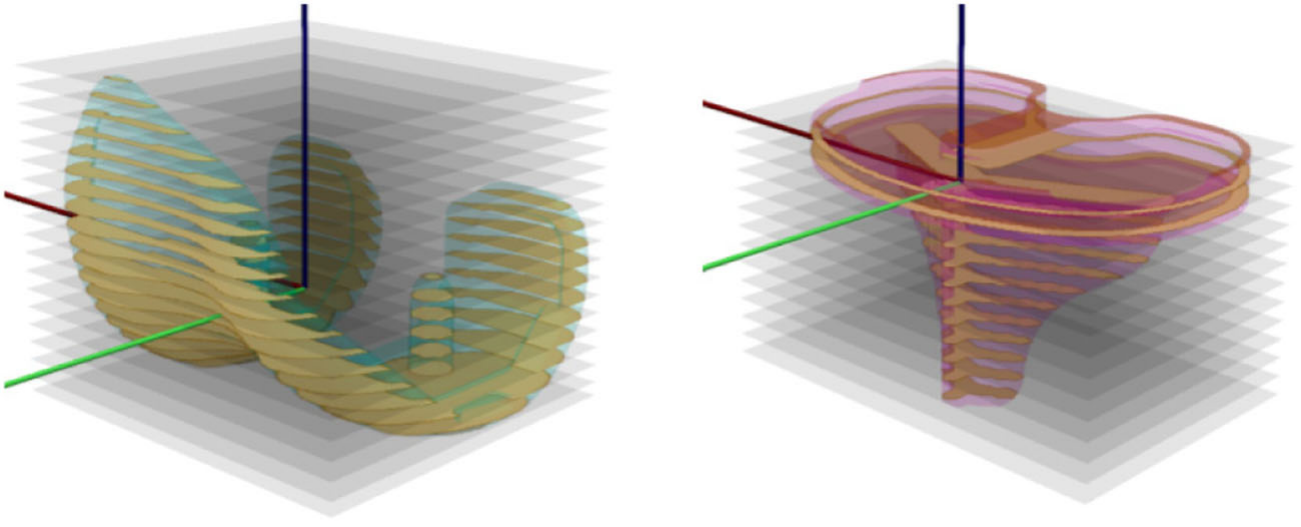
Illustration of the synthetic generated volumetric models. The voxels with the high intensity assigned value of 3000 are represented with the copper‐color at each slice. A transparent representation of the surface models is superimposed in each of the volume images to illustrate the outline of the models. Low‐resolution volumetric models (slice thickness of 3 mm compared with 0.4 mm) are presented for visualization. [Color figure can be viewed at wileyonlinelibrary.com]

The purpose of RSA was to find 3D spatial measures from 2D projective images to a 3D object. One marker‐based (marker) method represented the gold standard of RSA, and four model‐based (*mbrsa*, *autorsa‐surface*, *autorsa‐volume*, and *autorsa‐bone*) methods were evaluated (Table 1). We utilized manufacturer‐provided computer‐aided design models representing the 3D surface models of the femur and tibia implants in all sizes for three of the model‐based methods (*mbrsa*, *autorsa‐surface*, and *autorsa‐volume*). For the *autorsa‐volume* method, we constructed a synthetic volumetric representation of each implant model by assigning the voxels in a 3D isometric volume image inside the surface of the implant model to a value of 3000 and outside values to zero (Figure 2). The volume images with voxel spacing of 0.4 × 0.4 × 0.4 were centralized, oriented, and dimensioned according to the implants coordinate systems and bounding boxes using visualization Toolkit (Kitware). For the fourth method (*autorsa‐bone*), we obtained volumetric image models from the preoperative CT‐scan. Each bone model were identified and extracted individually using a fully automated graph‐cut segmentation method.^22^, ^23^, ^24^

**Table 1:** Overview of the radiostereometric analysis methods

Name	Software	Method	Description	Illustration
*marker*	MBRSA	Hugh circle detector	The actual marker projections were detected using Hugh circle detector based on manually applied parameters. **Marker‐method**: utilizing the actual markers on the stereoradiographs to estimate a 3D position of each marker by minimizing the crossing‐line‐distance of the marker for each view. **Marker‐configuration model**: utilizing a predefined 3D marker‐model estimating its 3D pose by minimizing the difference between its projected marker positions and the actual markers on the stereoradiographs.	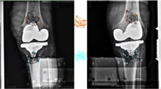
*mbrsa*	MBRSA	Canny edge detector	Contours were automatically detected in the stereoradiographs by canny edge detection based on manually applied parameters and region of interest. From these the relevant contours of the object of interest were manually selected. Coarser to finer optimization algorithms were applied to estimate the poses by minimizing the error between the virtual projections of the bone models and the manually selected contours.	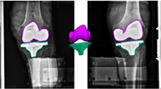
*autorsa‐surface*	AutoRSA	Silhouette‐projection	This automated image registration method utilizes silhouette‐projection of surface models that are matched to the actual object silhouette‐projection using Sobel edge detection algorithm. The similarity metric was determined as the average of the horizontal and vertical gradients normalized cross‐correlation between the actual stereoradiographs and the virtually generated projections. Coarser to finer optimization algorithms and image resolution were applied and an automated mask image were used to determine region of interest.	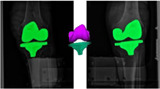
*autorsa‐volume*	AutoRSA	Digitally reconstructed radiographs	This automated image registration method utilizes digitally reconstructed radiographs of synthetic generated volumetric models that are matched to the actual object stereoradiographs using Sobel edge detection algorithm. The similarity metric was determined as the average of the horizontal and vertical gradients normalized cross‐correlation between the actual stereoradiographs and the virtually generated projections. Coarser to finer optimization algorithms and image resolution were applied and an automated mask image were used to determine region of interest.	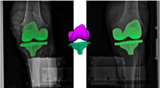
*autorsa‐bone*	AutoRSA	Digitally reconstructed radiographs	This automated image registration method utilizes digitally reconstructed radiographs of CT‐based volumetric models that are matched to the actual object stereoradiographs using Sobel edge detection algorithm. The similarity metric was determined as the average of the horizontal and vertical gradients normalized cross‐correlation between the actual stereoradiographs and the digitally reconstructed radiographs. Coarser to finer optimization algorithms and image resolution were applied and an automated mask image were used to determine region of interest and excluding the other implant.	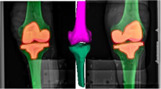

Abbreviations: AutoRSA, AutoRSA software, Orthopedic Research Unit, Aarhus, Denmark; CT, computed tomography; 3D, three‐dimensional; MBRSA, Model‐Based RSA (RSAcore, Leiden, the Netherlands); RSA, radiostereometic analysis.

We performed individualized calibration on all stereoradiographs by identifying the fiducial and control markers embedded in the calibration cage using the commercially available software Model‐Based RSA (RSAcore). The fiducial markers were used to calculate the focal points of the X‐ray sources. All methods were analyzed using the same stereoradiographs making the calibration identical between methods. Hence, the observed differences in displacement between methods was not influenced by the calibration. Model‐Based RSA was also applied for the displacement analysis using the inserted tantalum beads (*markers*) and the established semi‐automated model‐based (*mbrsa*) method.^25^

#### 2.4.1 Marker‐based method

The bone‐inserted markers at femur and tibia were identified in all images. To accommodate the recommendation of the RSA guidelines, we accounted for 0.35 mm as the upper limit of the mean rigid body error matching and ensured identification of the same markers in all images for one displacement series.^26^ The position was estimated as the centroid of the marker positions (*markers*).

#### 2.4.2 Model‐based method

Based on manually applied parameters and region of interest, the contours were automatically detected in the stereoradiographs by the Canny Edge Detector (RSAcore). The contours for the femur and tibia implants were then manually selected from these detected contours. Coarser to finer algorithms (IIPM, DIFDHSAnn, and DIFDoNLP) were applied to estimate the model pose by minimizing the error between the virtual projections of the models and the manually selected contours.^27^ (*mbrsa*) An effort was made to identify as much of the implant silhouette as possible.

#### 2.4.3 Investigated methods (AutoRSA software)

We investigated two methods that implemented a pin‐hole camera model to accommodate perspective projection like the established methods. One, utilized silhouette‐projection of a surface model, and one, utilized DRR projection of a volume model. Both methods used ray‐casting. While the surface‐based (*autorsa‐surface*) method only determined the model silhouette, the volume‐based (*autorsa‐volume* and *autorsa‐bone*) method estimated the DRR by calculating the ray's cumulative attenuation by each voxel it passed through the image volume. Image registration processes were accelerated utilizing the graphics processing unit (GPU) for speed improvement.

**Figure 3:**
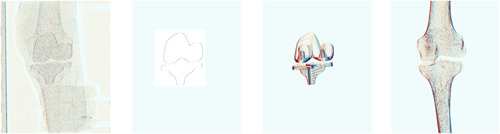
Horizontal Sobel gradient images of the left view. From left: actual radiograph, surface implant projection, digitally reconstructed radiograph implant projection, and digitally reconstructed radiograph bone projection. [Color figure can be viewed at wileyonlinelibrary.com]

**Figure 4:**
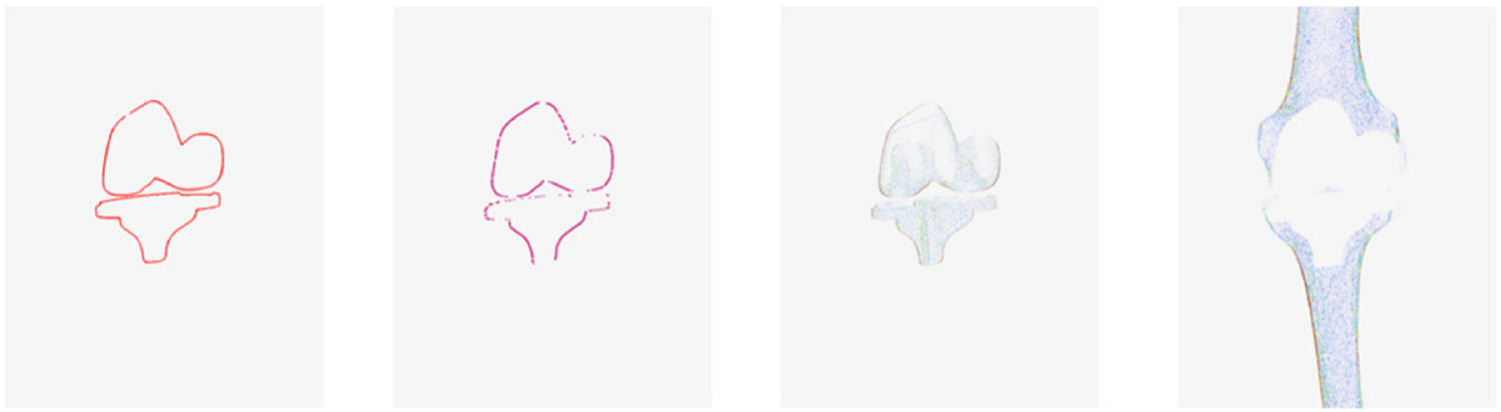
From left Model‐Based RSA implant contour detection, correlation image between the actual radiograph and surface implant projection for the horizontal Sobel gradient images, correlation image between the actual radiograph and implant digitally reconstructed radiographs for the horizontal Sobel gradient images, and correlation image between the actual radiograph and bone digitally reconstructed radiographs for the horizontal Sobel gradient images. Only the left image views are displayed. RSA, radiostereometic analysis. [Color figure can be viewed at wileyonlinelibrary.com]

For the 2D/3D registration purpose, according to previous metric evaluation and initial tests using CT based volumetric models, we found that the normalized gradient correlation worked best when comparing the virtually generated projections to the actual stereoradiographs.^28^, ^29^ The gradients were automatically determined using the Sobel edge detection algorithm.^30^ This algorithm calculated 2D spatial gradient measures at each pixel utilizing 3 × 3 convolution kernels, resulting in horizontal and vertical gradient images (Figure 3). The similarity metric was then determined as the average of the horizontal and vertical gradients normalized cross‐correlation between the actual radiographs and virtually generated projection (Figure 4).

The software allowed application of a mask‐image to exclude part of the image from the registration process. This benefits the registration in case of nonrelevant high image contrasts, which influenced the registration during initial tests. Two methods were optional in combination or separate. A predefined fixed mask‐image excluding high intensity objects like metallic object, and a dynamic mask‐image ensuring only analysis of the region of interest. The dynamic mask‐image were automatically defined as the initial dilated model projection, and thereby exclude the remaining part of the image.

We applied the program's robust optimization scheme that included a two‐stage registration process using the implemented nonlinear optimization library NLopt (Steven G. Johson, Boston, Massachusetts). First, a global optimizer (Controlled random search algorithm with local mutations), and second, a refined registration using a local optimizer (Nelder‐Mead Simplex).^31^, ^32^ We used half resolution images during the global optimizer, and full resolution images with activation of the dynamic mask during the refined local optimizer.

For the *autorsa* methods we analyzed each model separately. First, the femur and then the tibial implant models were analyzed using the surface‐based (*autorsa‐surface*) and volume‐based (*autorsa‐volume*) methods. For both methods, we applied the dynamic mask during the refined local optimizer in full image resolution. Second, the bone models were analyzed using the volume‐based (*autorsa‐bone*) method in the same order. With information of the preanalyzed implants poses, we utilized the fixed mask to exclude the surgically removed bone and metallic part of the implant from the registration. The fixed mask was produced automatically by dilating the implant silhouette‐projection obtained from the previous implant‐analysis. The fixed mask was applied during the global optimizer in half resolution and a combination of the fixed and dynamic mask during the refined local optimizer in full resolution. A desktop computer with a quad‐core processor (Intel Xeon E5‐1620, 3.60 GHz), 8 GB of DDR4 RAM, and a dedicated GPU (GeForce GTX 960, 4 GB GDDR5) completed the registration of a single stereoradiograph in approximately 30 s for the *autorsa‐surface*, 40 s for the *autorsa‐volume*, and 85 s for the *autorsa‐bone* methods.

### 2.5 Reference frame alignment between systems

While positioning the fixture at the X‐ray tubes cross‐section, we meticulously oriented the fixture reference frame, defined by the displacements of the three axial micrometers as closely as possible to the RSA reference frame defined by the calibration cage. Even so, the coordinate systems were not completely aligned. To accomplish error‐investigation in one direction individually, we defined the axial direction according to a linear fit of the 25 marker‐displacements for each series individually. Then, the investigated position coordinate of the models, expressed in the RSA reference frame, were projected to the fitted micrometers axial direction.

### 2.6 Statistical analysis

Accuracy measures of the the RSA registration methods were presented in Bland‐Altman plots facilitating the presentation of mean bias and limits‐of‐agreement (LOA) with a significance level of 0.05. Each of the five methods: Marker‐based of bones (*marker*), contour‐based of implants (*mbrsa*), surface‐based of implants (*autorsa‐surface*), volume‐based of implants (*autorsa‐volume*), and volume‐based of bones (*autorsa‐bone*), were presented separately for the femur and tibia, respectively. Statistical differences were not investigated between methods. For this study, it is not relevant whether methods are statistically different from one and another. What is relevant, is the accuracy of the method in context of the clinical application. Even so, to accommodate visual overview of the methods, their mean bias and LOA are summarized graphically.

## 3 RESULTS

We documented 205 recordings on 8 specimens resulting in a total of 1640 recordings. This included displacement series of two views (AP and LM) in three directions (*x*, *y*, *z*) with one series consisting of 35 stereoradiographs. We excluded 7 series due to nonmethodology issues such as a missing image in the recording or unlabeled retakes, resulting in 1395 analyzed stereoradiographs in total.

**Figure 5:**
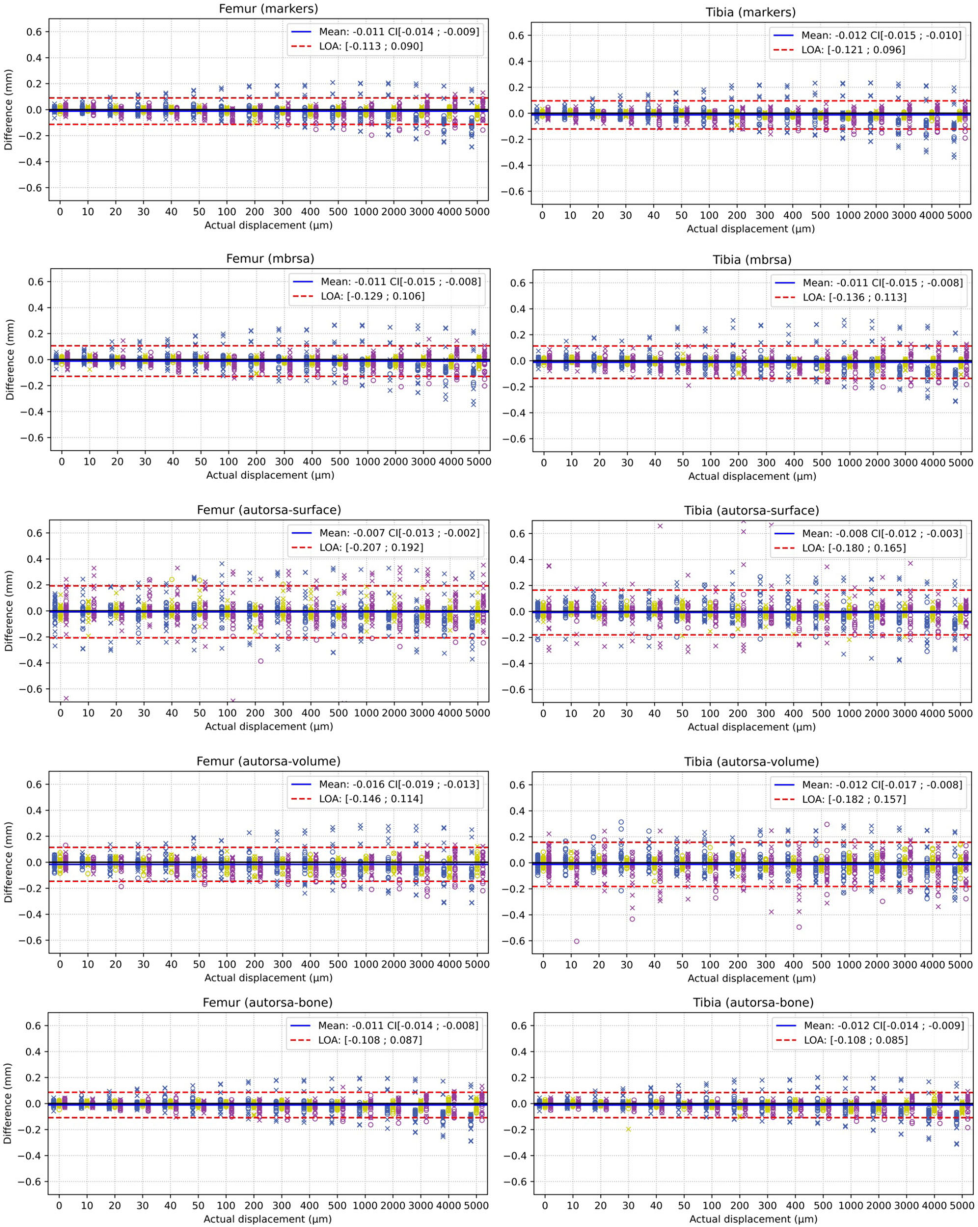
Bland‐Altman plots for each method with the colors representing the *x* (blue), *y* (yellow), and *z* (purple). The “o” and “x” represents the anterior‐posterior and lateral‐medial view, respectively. From top row: *marker*, *mbrsa*, *autorsa‐surface*, *autorsa‐volume*, and *autorsa‐bone*. The first column presents the data of the femur and the second column present data of the tibia. CI, confidence interval; LOA, limits‐of‐agreement. [Color figure can be viewed at wileyonlinelibrary.com]

**Figure 6:**
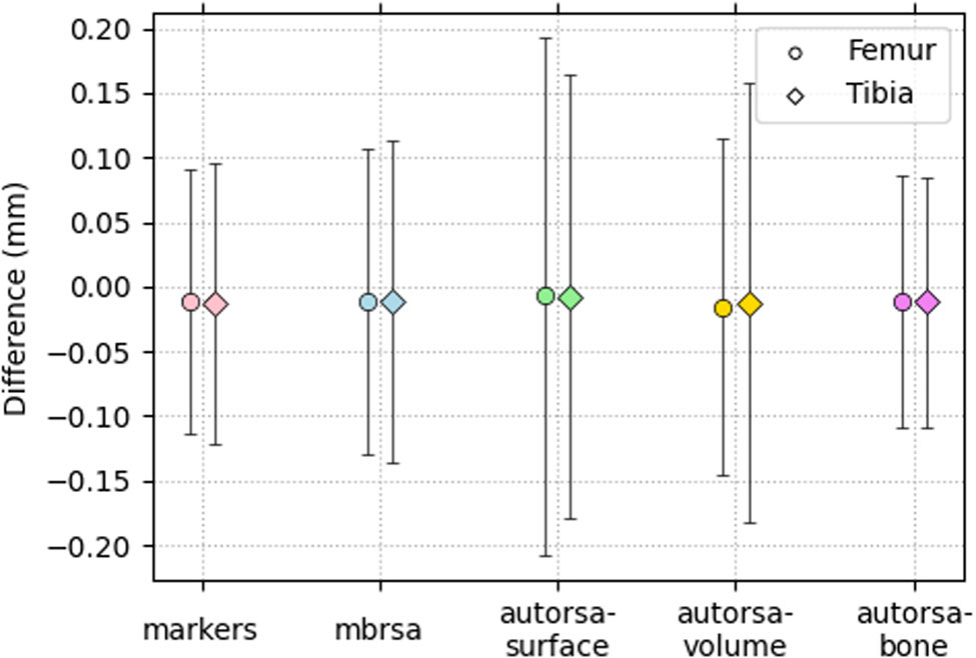
Presentation of the limit‐of‐agreements for each method for the femur and tibia, respectively. [Color figure can be viewed at wileyonlinelibrary.com]

The results of each registration method are presented as Bland‐Altman plots for the femur and tibia separately and revealed no bias for any of the analyzed methods (Figure 5). The best LOA was obtained for the marker and bone registration with similar results for both the femur and tibia. The worst LOA was found for the *autorsa‐surface* method for the femoral implant, while the *autorsa‐surface* and *autorsa‐volume* methods showed equally large LOA for the tibial implant. The *autorsa‐volume* method displayed similar LOA for analysis of the femoral implant as the *mbrsa* method. The *mbrsa* method displayed slightly worse LOA values than the marker‐ and bone‐methods (Figure 6).

For future power calculations, elaborated results of the individual directions separately for each view are presented in Tables 2 and 3, and data for the two views are combined in Table 4 for femoral and tibial models, respectively. In general, we found worse accuracy in the *z* direction for the AP view and in the *x* direction for the LM view, with the LM view being the worst. When the data for the two views are combined the tibia displayed in general slightly worst accuracy.

**Table 2:** Presenting the femur mean (standard deviation) of the different views in the three directions

	Anterior‐posterior view			Lateral‐medial view		
	*x*	*y*	*z*	*x*	*y*	*z*
*marker*	−0.027 (0.043)	−0.011 (0.018)	*−0.028 (0.049)*	***−0.002 (0.102)***	−0.001 (0.016)	−0.005 (0.041)
*mbrsa*	−0.027 (0.042)	−0.009 (0.015)	*−0.038 (0.052)*	***0.004 (0.123)***	−0.004 (0.019)	−0.003 (0.049)
*autorsa‐surface*	−0.027 (0.054)	−0.006 (0.031)	*0.036 (0.074)*	***−0.018 (0.161)***	0.001 (0.051)	0.026 (0.149)
*autorsa‐volume*	−0.023 (0.049)	−0.013 (0.033)	*−0.050 (0.070)*	***−0.000 (0.127)***	−0.009 (0.030)	−0.010 (0.049)
*autorsa‐bone*	−0.026 (0.040)	−0.010 (0.021)	*−0.027 (0.044)*	***−0.003 (0.098)***	−0.004 (0.018)	−0.002 (0.040)

*Note*: The italic numbers present the highest standard deviations for each method and view. The bold‐italic numbers present the highest standard deviation for each method in both views.

**Table 3:** Presenting the tibia mean and standard deviation of the different views in the three directions

	Anterior‐posterior view			Lateral‐medial view		
	*x*	*y*	*z*	*x*	*y*	*z*
*marker*	−0.026 (0.043)	−0.008 (0.013)	*−0.035 (0.046)*	***0.003 (0.114)***	−0.008 (0.015)	−0.006 (0.043)
*mbrsa*	−0.026 (0.045)	−0.008 (0.016)	*−0.031 (0.056)*	***0.011 (0.126)***	−0.009 (0.023)	−0.010 (0.059)
*autorsa‐surface*	*−0.020 (0.072)*	−0.003 (0.025)	−0.029 (0.058)	***0.007 (0.131)***	−0.010 (0.040)	0.004 (0.131)
*autorsa‐volume*	−0.006 (0.084)	−0.008 (0.039)	*−0.032 (0.107)*	***0.015 (0.120)***	−0.007 (0.025)	−0.035 (0.105)
*autorsa‐bone*	−0.022 (0.039)	−0.014 (0.020)	*−0.024 (0.041)*	***−0.002 (0.101)***	−0.010 (0.023)	−0.002 (0.034)

*Note*: The italic numbers present the highest standard deviations for each method and view. The bold‐italic numbers present the highest standard deviation for each method in both views.

**Table 4:** Presenting the combined mean and standard deviation of both views in the three directions for the femur and tibia, respectively

	Femur			Tibia		
	*x*	*y*	*z*	*x*	*y*	*z*
*marker*	*−0.015 (0.077)*	−0.006 (0.018)	−0.014 (0.046)	***−0.013 (0.085)***	−0.008 (0.015)	−0.005 (0.041)
*mbrsa*	*−0.012 (0.091)*	−0.006 (0.017)	−0.017 (0.053)	***−0.009 (0.094)***	−0.008 (0.020)	−0.018 (0.059)
*autorsa‐surface*	−0.023 (0.117)	−0.002 (0.043)	***0.002 (0.129)***	−0.008 (0.104)	−0.006 (0.034)	*−0.009 (0.110)*
*autorsa‐volume*	*−0.013 (0.094)*	−0.011 (0.031)	−0.026 (0.061)	−0.003 (0.102)	−0.007 (0.032)	***−0.034 (0.106)***
*autorsa‐bone*	*−0.016 (0.074)*	−0.007 (0.020)	−0.011 (0.043)	***−0.013 (0.075)***	−0.012 (0.022)	−0.010 (0.038)

*Note*: The italic numbers present the highest standard deviations for each method and bone. The bold‐italic numbers present the highest standard deviation for each method in both bones.

## 4 DISCUSSION

In this study, we evaluated the accuracy of an automated 2D/3D registration method of implant and bone models. Our study adds to previous knowledge with two key findings. First, we evaluated a marker‐free bone registration method for an arthroplasty knee joint where the radiopaque implant components occluded or replaced a large part of the bone. It provided similar accuracy compared to the gold standard marker‐based method. Second, we evaluate a synthetic volumetric implant model utilizing DRR, which provided more model information to improve registration in RSA. However, the volumetric implant model and DRR method, in its present form, did not markedly improve the silhouette‐projection method.

Various studies have proposed image registration methods for both single and dual radiographic focus systems.^9^, ^10^, ^11^, ^12^, ^13^, ^14^, ^15^, ^16^, ^17^, ^18^, ^33^, ^34^, ^35^ The single imaging system are subject to poor out‐of‐plane performance compared to in‐plane,^13^, ^33^ which can be overcome using a dual focus system.^12^, ^13^ When estimating accuracy the definition and establishment of ground truth is crucial. Ideally, the gold standard should be very accurate—at least as accurate as the method being tested, and hopefully much better, while being independent to the investigated method. Most image registration accuracy studies have been oriented towards dynamic studies making radiographic‐independent and submillimeter gold standard difficult. Kaptein et al.^7^ evaluated static images and used an approach similar to the *marker*‐ and *mbrsa*‐method demonstrating similar in‐plane results while our results were superior in out‐of‐plane direction. Reasons for these differences may be explained by different implants, better optimizers in the software, improved radiographic technology, and image size. Our automated methods showed overall similar or better results to those previously presented by Kaptein et al.^7^ when comparing the AP view of the specimens as they also used.

### 4.1 Partial bone registration in artificial joint

Bone‐implant interface fixation is considered an important predictor of long‐time outcome, and may provide useful information in patients with inexplainable pain and dysfunction of knee arthroplasty.^4^ Additionally, inducible micromotion RSA has shown promising results as an instant predictor of long‐term loosening, and thereby could eliminate the otherwise required time‐expensive follow‐up period. Simple RSA recording along with a CT scan provide information to evaluate inducible micromotion without the need for embedded tantalum markers. However, literature on image registration methods of bones within total knee arthroplasty joints are sparse. Seehaus et al.^19^ and Kim et al.^20^ presented bone registration in knees with TKA.^19^, ^20^ Their performance measure differs from the present study making comparison difficult. Moreover, Seehaus et al.^19^ showed migration precision results that exceeded the traditional marker‐precision to such a degree that they precluded feasibility of ‘completely markerless’ migration calculation in the presented form. Kim et al.^20^ presented implant and bone translation repeatability ranging between 0.019 and 0.142 and 0.030 to 0.289, respectively. In contrast to our results, Kim et al.^20^ found worse bone registration than implant registration. This may be explained by the applied registration methods. They utilized silhouette‐projection and canny‐edge detection that did not utilize the valuable intensity information of the bone.^9^ Additionally, the optimizer may be inhibited to reach the global optima, as the preoperative articulating surface part of the bone silhouette‐projection and the postoperative articulating surface part of the actual implant may coincide. Contrary, we utilized bone intensities from DRR registration and eliminated negative influence of the implant by taking advantages of the known implant projection from the precompleted implant registration.

### 4.2 Synthetic generated volumetric implant model

This study showed that inadequate gradient information was present within the actual stereographs to take advantage of the enhanced information from the DRR. It may be due to the radiopaque nature of the implants. However, the correlation images display that some information were included in the similarity metric, and we saw some improvement in accuracy for the femoral implant in the LM view. Visual inspection of the stereographs also clearly showed intensity differences; thus, we speculate that other similarity metrics may provide improved accuracy. Previously, Mahfouz et al.^35^ presented a similarity metric combining gradient images and the radiographs, and Scarvell et al.^36^ presented a similarity metric using cross‐correlation residual entropy (CCRE) of intensity‐based edge‐enhanced images. CCRE is a mutual information measure that benefits image registration where identical image intensity cannot be presumed, and this is the case for the actual radiograph and the estimated DRR. Continuous research is needed to investigate other similarity metrics.

### 4.3 Limitations

First, establishing a gold standard for accuracy measurements, which is not affected by the radiographic methods is challenging. We used a micrometer to estimate the displacement between recordings; however, a baseline estimate of the zero position was still required. To avoid including other registration methods, we used the median of five recordings to estimate a solid baseline and a clinical applicable migration accuracy. With no proportional bias we believe that five recordings were sufficient. Second, we used a single implant design. Other TKA designs may influence the accuracy of the method. A more unique design of the implant will be easier to register than a symmetric implant. A difference in implant size will also influence the method accuracy, the bigger the implant the better the results will be. Similar influence of the shape and size of the joint may also affect the accuracy. Third, we used marker‐data to establish the alignment between the fixture and the RSA system and therefore may induce uncertainty in the directions. However, only small differences between the orientation of the systems were identified, and we applied the same alignment for each series to all methods ensuring a fair comparison. Fourth, we applied a preoperative CT volumetric bone model without potentially implant artifacts which would be present within a postoperative CT volume. Our experience of postoperative stereoradiographs and the areas of metal artifacts in the CT volume is that these artifacts are anticipated to be hidden behind the implant using the traditional anteroposterior view for the stereoradiographs. Furthermore, mandatory preoperative CT may increase with a direction towards robotic and personalized TKA surgeries.^37^, ^38^, ^39^ Finally, it should be noted that this study does not provide direct information on the rotational accuracy in a static setup—only translational accuracy in different views were assessed and the results may not provide equally high accuracy for a dynamic setup.

### 4.4 Conclusion

In conclusion, marker‐free RSA is feasible, with an automated 2D/3D image registration method utilizing CT obtained volumetric bone models and DRR (*autorsa‐bone*), within a similar accuracy as achieved with the gold standard marker‐based RSA (*marker*). Furthermore, an automated 2D/3D image registration method utilizing synthetic generated volumetric implant models (*autorsa‐volume*) could not improve the silhouette‐based method (*autorsa‐surface*). The automated registration methods exhibit feasible accuracy in relation to the present knowledge within the literature, however, the semi‐automated canny‐edge detection method (*mbrsa*) exhibit slightly better accuracy.

## AUTHORS CONTRIBUTIONS

**Emil Toft Petersen**: was involved in all aspects of the study and drafted the manuscript. **Tobias Dahl Vind**: had an essential role in the RSA analysis. **Jonathan Hugo Jürgens‐Lahnstein, Rasmus Christensen, and Søren Rytter**: assisted in data acquisition, data analysis, and interpretation of data. **Sepp de Raedt**: assisted in study design and data interpretation. **Annemarie Brüel**: contributed to the study design and acquisition of cadaveric specimens. **Michael Skipper Andersen**: had an essential role in interpretation, and presentation of data. **Maiken Stilling**: had an essential role in the study design, interpretation, and presentation of data. All authors critically revised and approved the final manuscript.

## CONFLICT OF INTEREST

The authors declare no conflict of interest.
